# Determinants of adoption of urban agricultural practices in eastern Haraghe zone of Oromia region and Dire Dawa City administration, eastern Ethiopia

**DOI:** 10.1016/j.heliyon.2024.e26758

**Published:** 2024-02-20

**Authors:** Gebregziabher Nigus, Mengistu Ketema, Jema Haji, Million Sileshi

**Affiliations:** a School of Agricultural Economics and Agribusiness, Haramaya University, Haramaya, Ethiopia; b Ethiopian Economics Association, Addis Ababa, Ethiopia

**Keywords:** Urban agriculture, Adoption, Determinants, MVP model, Ethiopia

## Abstract

Although urban agriculture (UA) can aid economic development, food inflation, unemployment, and nutritional insecurity often necessitate urban households to engage in various agricultural practices. Hence, the study aimed to identify UA practices adopted and their determinants in the Eastern Haraghe zone of the Oromia region and Dire Dawa City Administration, Eastern Ethiopia. Data was collected from 385 randomly selected urban households using a semi-structured questionnaire. Descriptive statistics and a Multivariate Probit (MVP) model were used to analyze the data. The results of the MVP model reveal that sex, age, education level, land size, extension contact, credit access, community group participation, training, and household non-farm income significantly influenced vegetable farming adoption. On the other hand, livestock production adoption was also affected by factors such as sex, land size, perception, credit access, farming experience, community group participation, market distance, training, and non-farm income. Crop-fruit production adoption was also influenced by factors like age, perception, farming experience, market distance, training, and dependency ratio. Improving the ability to use the land for UA purposes, empowering female-headed households, improving livestock breeds, creating awareness through short-term training, and improving credit accessibility are important recommendations to enhance the adoption of UA practices in the study areas.

## 1 Introduction

The global urban population has risen rapidly, leading to a mount in food insecurity among the urban destitute [1]. This concern is especially pressing in developing nations, notably in Sub-Saharan Africa (SSA). As a result, guaranteeing urban inhabitants' food security has become a crucial concern, with the emphasis shifting from rural to urban regions [2]. Food insecurity has a detrimental impact on households and individuals, as efforts to achieve it might be costly. For instance, acute food and nutritional insecurity may lead vulnerable households to psychosocial dysfunction, socio-familial problems, depression, and poor health [[3], [4], [5]].

Food insecurity in SSA urban areas must be addressed to meet parts of the United Nations' sustainable development goals, notably the objective of reaching “zero hunger” and “zero poverty” [2]. One mechanism being considered as a solution is encouraging agricultural practices within urban and peri-urban limits. Urban agriculture (UA) is a reaction to food insecurity, particularly among the impoverished, and it involves employment creation, revenue generation, and environmental protection. It is critical to supply fresh, affordable, and healthy food to urban dwellers [6,7]. UA is the practice of growing crops, vegetables, and fruits and rearing small livestock or milk cows at the household level for consumption or sale within urban and peri-urban settings [8].

The growth of town/city, along with urban food insecurity, substantially influences the need for resources such as sustainable infrastructure and inexpensive food retail alternatives to meet the fundamental needs of families living in city/town bounds [9]. The cost of supplying, distributing, and accessing food is anticipated to rise as the number of food-insecure urban dwellers grows [10]. Due to financial and physical limitations, many urban dwellers are unable to obtain the food they need. Therefore, the implementation of UA can be taken as one measure for combating urban food insecurity [11]. In line with this, there have been encouraging developments recently in Ethiopia. The adoption of UA has become increasingly popular and is being promoted to sustain the lives of poor and unemployed urban dwellers [12]. In Ethiopia, UA has long been practiced, with households keeping livestock, sheep, and poultry or growing rain-fed crops and vegetables on plots adjacent to their homes [13]. UA is expected to provide a significant percentage of the town's/city's need for eggs, poultry, dairy products, and green vegetables. Municipal officials in the study areas have begun to implement UA as a food security strategy following the “Yelemat-trufat” initiative in response to food self-sufficiency at the family and national levels. Despite this intervention, there is little information on UA practice in the country.

Addressing UA necessitates multifaceted approaches and a thorough understanding of the factors contributing to or impeding the sub-sector. So far, the most outstanding UA literature has been through systematic reviews focused on narrating the contribution and constraints. For example, UA in Ethiopia [14], urban food systems [15,16], livelihood and food security [17,18], and practices and barriers to sustainable UA [[19], [20], [21]] which have mainly focused on the general view of UA status in an urban context. Unlike these [22,23], have sought to explore factors influencing UA practices. However, the study of [22] was limited in terms of coverage and method. The study utilized a simple extension of the binomial logistic regression model (multinomial logit model) that does not account for potential interaction amongst UA practices and is based on a single town instance. In addition, the study only looked at crops and livestock practices, leaving out vegetable practices, which are the most popular in urban. A similar argument can be made for [23] study findings, which used the logit model to detangle all forms of UA practices. The study focused on those who commercialize UA, where the constraints at the household level are more important than those who commercialize it because commercial farmers are expected to have the financial capability to overcome the associated problems.

A previous study on UA has been limited, and there are no empirical research outputs on socioeconomic, demographic, and other associated factors that impact UA practices in Ethiopia in general and the eastern Haraghe zone of Oromia and the Dire Dawa city administration in particular. Although the local government increased its efforts to expand the sector recently, UA is getting much more complicated owing to a lack of empirical evidence on the decisive factors impacting it. There is a lack of case-specific studies that depict UA and the main factors that affect it. The insufficiency of research evidence on the factors that influence UA practices makes it challenging to develop a plan of action and combat its limitations. To that end, knowing the local UA settings while considering socioeconomic, demographic, and institutional issues into account is critical for local governments and donors to act and strengthen the subsector and thereby encourage urban dwellers to reap the benefits that it offers. Moreover, the association between the various UA practices must be thoroughly investigated, documented, and shared. Hence, this study contributes to the existing literature by identifying the determinants of UA practices using a multivariate probit model while accounting for the interdependence of each UA practice, which was not addressed in previous studies. Additionally, provide local authorities and other interested organizations with empirical evidence so they may use it as input in their initiatives to expand the sector across similar urban settings.

## 2 Methodology

### 2.1 Description of the study areas

Descriptive statistics and an econometric model called MVP model are used to analyze data. The study is conducted in the Dire Dawa city administration, one of the two city administrations, and the eastern Hararghe zone of Oromia, found in eastern Ethiopia. Dire Dawa is located 515 km away from Addis Ababa, the capital city of Ethiopia, and 55 km north of Harar [24]. Geographically, Dire Dawa lies between 9°27'N to 9°49'N latitude and 41°38'E to 42°19'E longitude. Whereas, the east Hararghe zone capital Harar is located 510 km to the east of the Ethiopian capital, Addis Ababa. Its altitude ranges from 500 to 3,400 m above sea level. Geographically, the east Hararghe zone lies between coordinates of 8° 48'28.9008'' N latitude and 41°36'4.2516'' E longitude. The zone contains three agro-ecological zones, Dega (highlands elevations above 2,300 m), Woinadega (midlands elevations between 1,500 and 2,300 m), and Kola (lowlands below 1,500 m). The Kola (lowlands) occupies the largest area (62.2%), followed by Woinadega (26.4%) and Dega (11.4%) [25].

The east Hararghe zone encompasses several small towns; the major ones are eleven, including Harar City. Demographically, the zone has a total population of 3,538,361, While 350,014 or 9.89% are urban inhabitants, the remaining 3,188,346 or 90.11% are rural inhabitants [26]. According to the same report, Dire Dawa city administration has a total population of 466,000. While 293,000 or 62.88% are urban inhabitants, the remaining 173,000, or 37.12% are rural inhabitants.

Urban agriculture encompasses various livelihood systems in the study areas, ranging from subsistence production and processing at the household level to commercial agriculture. It takes place in different locations and under varying socio-economic conditions with various production systems involving a diversity of interdependent vegetable, fruit, crop production, and livestock-raring activities. The agro-climatic zone stretches from the lowland (Dire Dawa city) to the highland (Kulubi town), which enables the production of a range of agricultural products including cereal crops like sorghum and maize; vegetables like potatoes, tomatoes, onions, salad, green peppers, and cabbage; fruit trees like oranges, mangoes, bananas, lemons, and papayas; and perennial crops like coffee and khat (Catha edulis) [27]. The areas are also convenient for livestock raring, such as milk cows, cattle beef fattening, goats, sheep, poultry production, and beekeeping activities.

### 2.2 Sample size and sampling technique

Household heads were considered key decision-makers and respondents for UA practices in the study. A multi-stage method of sampling was employed to choose the target sample households. First, the east Haraghe zone and Dire Dawa City Administration were specifically chosen for their relevance to the study's objectives. These study areas were specifically chosen because they comprise a large number of towns and communities that engage in diverse UA practices on a variety of scales. In the second stage, Dire Dawa city and four towns from the east Haraghe zone of Oromiya were selected based on the existence of UA practices (major UA-practicing towns in the zone) and discussions held at the zone’s office of agriculture. In the third stage, Kulubi, Kersa, and Haramaya towns as a whole, four kebeles from Harar and four kebeles from Dire Dawa city, were randomly selected for the study. Finally, 385 sample household heads were randomly selected and interviewed from each town/city, proportionate to the size. The study regarded the head of the household as the primary respondent since the head is responsible for making economic decisions for the family.

### 2.3 Data types, sources, and data collection methods

This study relied primarily on primary data sources; other relevant secondary data were also collected to supplement the data. Face-to-face interviews with individuals utilizing semi-structured questionnaires were used to gather primary data. The data collection period lasted from July 2022 to August 2022. Published and unpublished sources, such as from the Bureau of Zonal Agriculture as well as CSA reports were among the secondary data sources collected. When preparing the questionnaire, previous studies on the factors influencing the adoption of agricultural practices were taken into consideration [22,23,28]. In this study, a mixed research strategy was adopted since a single approach of research method, either qualitative or quantitative, may not be sufficient to comprehend the important limitations in UA practices. As a result, quantitative approaches should be complemented by qualitative methodologies [29]. Accordingly, focused group discussions (FGD) and Key Informant Interviews (KII) were conducted in each kebele to enrich and corroborate the survey results. KII was conducted with experts from zonal and district offices of agriculture and town/city development agents.

### 2.4 Methods of data analysis

The correlation contained in the error terms of the adoption equation is not taken into account by univariate models like Probit and Logit. Therefore, applying such models is inappropriate when the adoption of agricultural practices is interrelated [30]. [31] suggests that inefficient and biased estimates may be obtained by not considering unobserved factors and the interdependent relationship between adoption decisions on different UA practices. Empirical studies show that agricultural practices are interdependent, and the adoption of one practice can influence the likelihood of adoption [[32], [33], [34]]. As a generalization of the Probit model, the multivariate Probit (MVP) model allows for the simultaneous estimation of many correlated binary outcomes [35]. The MVP estimation approach analyses the influence of factors on urban farming practices and estimates a series of binary Probit models to assess the correlation in error terms. It establishes the relationship between the adoption of different UA practices and potential correlations between unobserved disturbances [30].

The study identified three main UA practices (i.e., vegetable production, livestock rearing, and crops and fruits production), and households were more likely to jointly adopt a mix of these techniques to deal with their production constraints than to adopt a single practice. Therefore, this study applied the MVP model because it concurrently predicts the impact of the explanatory factors on each dependent variable while enabling the unobserved characteristics to be freely correlated. When adoption choices are made simultaneously, modeling them using an MVP framework improves estimation accuracy [36]. The model may be described empirically as follows:

(1) $$Y_{jpk}^{*}=\beta_{k}X_{jp}+X_{j\alpha k}+\varepsilon_{jp}\;\left(K=V,L,C\right)$$

Where $X_{jp}$ represents observed characteristics; ($X_{j}$) represents the mean value of covariates; ($\varepsilon_{jp}$) represents a multivariate normally distributed stochastic terms; K denotes the type of UA practice [representing the choice of vegetable production (V), livestock raring (L), and crop & fruit production (C)] and $\beta_{k}$ denotes the vector of the parameter to be estimated.

For each choice, the unobserved preferences in Eq. (1) translate into the observed binary outcome equation as follows [37]:

(2) $$Y_{k}=\left\{ \begin{array}{c} 1\;if\;Y_{jpk}^{*}\;>\;0\\ 0\;otherwise\; \end{array}\right.\;\left(K=V,L,C\right)$$

The error terms jointly follow a multivariate normal distribution (MVN) in the MVP model [37], where $\left(u_{V},u_{L},u_{C}\right)$ ∼ MVN(0, Ω) and the symmetric covariance matrix Ω is provided by:

(3) $$\Omega =\left[\begin{array}{ccc} 1 & \rho VL & \rho VC\\ \rho LV & 1 & \rho LC\\ \rho CV & \rho CL & 1 \end{array}\right]$$

A pairwise correlation coefficient (ρ_ij_) is used to estimate the model's error terms, with off-diagonal elements denoting unobserved correlations between stochastic components of distinct practice types.

### 2.5 Ethical consideration

Approval to conduct research and collect data from respondents was obtained from Haramaya University Post Graduate Research with a research permit of HUSP_2022_3408. Furthermore, respondents were asked to provide voluntary consent before taking part in the interview.

## 3 Results and discussion

### 3.1 Socio-demographic characteristics

The summary statistics of the socioeconomic characteristics of the sample households were determined and provided in Table 1. Adoption in this study is measured by the proportion of households practicing livestock raring (such as poultry, dairy cows, goats, sheep, and/or beekeeping), and the cultivation of vegetables, crops, and fruits in urban and peri-urban areas for own consumption or sale. According to the descriptive test result, adopters are distinguishable in terms of household characteristics such as sex, age, education, occupation, family size, credit access, farming experience, participation in a community group, non-farm income, perception of the economic benefits of UA practices, and participation in any UA-related training.

**Table 1:** Summary statistics of continuous and discrete variables.

		Adopters (270)		Non-adopters (115)		Total (385)	
Variables	Description	Mean	SD	Mean	SD	Mean	SD
Sex	Sex of household head (1 if female)	0.711***	0.454	0.530	0.501	0.657	0.475
Age	Age of the household head in years	41.944***	11.173	45.409	11.834	42.979	11.469
Education	Education level (years of schooling)	10.911***	4.102	8.496	4.903	10.19	4.489
Occupation	Occupation (1 if civil servant)	0.533***	0.500	0.730	0.446	0.592	0.492
Family size	Household size	5.544***	1.864	6.061	1.948	5.699	1.902
Land size	Land size (in hectares)	0.234	0.259	0.167	0.223	0.214	0.250
Extension	Extension contact (in number)	0.874	1.352	0.774	1.271	0.844	1.327
Perception	Perception about benefits of UA (1 if yes)	0.574***	0.495	0.026	0.160	0.410	0.493
Credit	Access to credit (1 if yes)	0.289***	0.454	0.200	0.402	0.262	0.440
Experience	Farming experience (in years)	10.874***	7.306	6.757	7.205	9.644	7.507
Participation in a Community Group	Member of a community group (1 if yes)	0.863***	0.345	0.243	0.431	0.678	0.468
Market Distance	Market Distance (in Km)	2.516	1.388	2.343	1.196	2.464	1.335
Training	Participated in UA-related training (1 if yes)	0.715***	0.452	0.061	0.240	0.519	0.500
Dependency ratio	Dependency ratio	0.325	0.351	0.293	0.305	0.315	0.338
Non-farm income	Non-farm income (in Birr^a^)	57.58k**	13.59k^b^	54.79k	16.55k	56.75k	14.57k

a The exchange rate at the time of data collection was 1$ for 52 birr.

b K stands for thousand birr.

The sample households in the study areas are female-dominated, as evidenced by 65.7% of female household heads (Table 1). The findings also showed that adopters (71.1%) had considerably more female household heads than non-adopters (53%). During the KII, participants noted that there is minimal obvious usage of wage labor in the UA subsector. As a result, informal work in UA might give supplementary opportunities to underemployed females where formal sector prospects are limited. The average age of the head of the household in the study area was 42.98 years. For adopters, the average age of the respondents was 41.94 years old, but for non-adopters, it was 45.41 years old. The test statistics findings revealed that there were significant age disparities between adopters and non-adopters. In terms of the relationship between household age and UA practice choice, the data showed that older urban farmers were active in livestock rearing, whereas most young urban farmers practiced both crop-fruit and vegetable production.

The sample households had a mean family size of 5.7 persons. The average family size for adopters and non-adopters was 5.54 and 6.06, respectively. Adoption of UA practices is higher in small families than in large families, as evidenced by significant differences at the 1% probability level. This might be related to the time required for UA practices; households with large family sizes dedicate most of their time to nursing their children, which may jeopardize the labor needs of UA practices. The mean dependency ratios were 0.33 and 0.29 for adopters and non-adopters, respectively. This reveals that compared to non-adopters, adopters supported a greater number of individuals who were either very young or very old.

In comparison to non-adopters, adopters' household heads had higher average levels of education (years of schooling), which gave them a better understanding of the value of adopting UA practices. According to the findings, household heads who were UA adopters had an average age of 10.91 years, while those who were not had an average age of 8.5 years. When it comes to the occupations of the sample household heads, 59.2% of the sample respondents were civil servants. In terms of adopters and non-adopters, the proportions of civil servants were 53.3% and 73%, respectively. There is a significant association between UA practices adoption and occupation of the household head at 1% level. In terms of the relation between the choice of UA practices and occupation, the data indicate that the majority of civil servants used to practice vegetable production.

In UA activities, non-farm income and land are crucial productive assets. The results of the non-farm income showed that there was a significant difference between UA practice adopters and non-adopters. The average non-farm income of adopters was 57.58 thousand birr, while it was 54.79 thousand birr for non-adopters. The mean land size of UA practices for adopters and non-adopters was 0.23 ha and 0.17 ha, respectively. The average distance from a market center for adopters and non-adopters was 2.52 km and 2.34 km, respectively. The data show a mixed result when it comes to UA practice choice and land size; those who own relatively large land size and have better non-farm income used to practice crop-fruit production and livestock rearing.

Credit is an essential institutional component influencing livelihood strategy choices. It is also an important component of self-employment in the agricultural, off-farm, and non-farm sectors [38]. Households who have access to credit do not have financial constraints to purchase production inputs, use improved technologies, and expand income-generating activities. The descriptive statistics result indicated, that 26.2% of the respondents have access to financial institutions and received various amounts of credit. The proportion of adopters having access to credit institutions was 28.9%, while it was 20% among non-adopters. In terms of UA practice choice, the majority of households with access to credit tend to practice livestock rearing. The mean value of extension contact per year for sample households was 0.84. The mean value of extension contacts per year of adopter and non-adopter households was 0.87 and 0.77, respectively. Moreover, the proportion of sample households who participated in UA-related training was 51.9%. There was a significant difference in the proportions of adopters and non-adopters, which were 71.5% and 6.1%, respectively.

The sample households expressed their perception of the different attributes of UA practices regarding food security, income, and employment opportunities of the UA practices. Accordingly, around 41% of the respondents perceived that UA is beneficial. In terms of adopters and non-adopters, the proportion was 57.4% and 2.6%, respectively. Moreover, among the total sample households, 67.8% were members of a community group, while the proportion in terms of adopters and non-adopters were 86.3% and 24.3%, respectively. The mean farming experience of adopters was 10.87 years, whereas, for non-adopters, it was 6.76 years. The test findings also revealed significant variations between adopters and non-adopters.

### 3.2 Constraints of urban agriculture practices

Despite all of the benefits it provides, UA practices are not without challenges or constraints. Sample household's responses on the constraints of UA production are listed in Table 2. These days, insufficient land is one of the most restricting resources facing urban and rural Ethiopian households [39]. Limited access to land was the primary problem pointed out by the majority of survey respondents (77.40%). Especially for those who use open and roadside spaces for UA practices, lack of ownership of land was a concern. During the FGDs, respondents highlighted that due to the shortage of land, the most affected were the marginalized groups and minorities, who have the greatest need to engage in UA.

**Table 2:** Constraints of urban agriculture practices.

No	Constraints	Frequency	Percent
1	Lack of land for production	298	77.4
2	Lack of inputs	65	16.88
3	Lack of improved breeds	17	4.42
4	Water shortage	185	40.05
5	Pest and disease stress	72	18.7
6	Treat of pollution	31	8.05
7	Poor access to information and agriculture extension services	46	11.95

Water scarcity, like that of land, is a challenge to practicing UA in the study towns/cities. Water is not only one of the most essential resources for UA practices but also in high demand and growing scarce in the region (especially in Harar and Dire Dawa cities) [40]. The need for clean drinking water is still in great demand, and its greatest value is not for use in agriculture. So, among the problems preventing the implementation of UA practices are water shortage and pollution. Respondents indicated that lack of water (40.05%) or drought conditions were their most serious problems. For instance, some urban farmers in the study towns/cities utilize costly potable water from the town/city water supply, some have deep wells built up, and others use contaminated water from nearby factory outlets (like the Harar Brewery). During the KII and FGD, participants pointed out lack of water use threatens farming activities and urban development.

Participants noted the presence of pests and weeds had an adverse effect on UA production. To overcome the problems, urban farmers utilize biological or cultural pest control methods and chemicals such as DDT, which have serious health repercussions. Respondents also identified input constraints (like seeds and fertilizers), pest sides, selected breeds, animal feeds, etc. According to FGDs and KIIs, the cost of inputs (especially prices of seed, medication, and animal feed) is increasing over time. Animals owned by households were unable to receive the required medical care. Particularly, the majority of livestock owners experienced illness and cattle mortality. Most of the time, medication was administered privately and without a professional diagnosis. A study conducted by Ref. [41] supports this finding. They discovered a number of prevalent ailments, such as diseases of the skin, bloating, anthrax, black legs, and both internal and external parasites. Moreover, the threat of pollution was another concern mentioned, particularly for those who sourced their irrigation water from industrial operations.

### 3.3 Empirical results of the determinants of adoption of urban agricultural practices

A total of 15 explanatory variables are included in the model of which 13 variables are significant in determining the adoption of at least one of UA practices. The model’s Chi-square test result is statistically significant at less than 1% level, indicating that the explanatory variables taken together are relevant in explaining the variability in UA practices adoption in the study areas (Wald ***χ***2 (45) = 711.07, P = 0000); this indicated that the overall fitness of the model. The likelihood ratio test rejects the null hypothesis that the UA practices adopted are independent (***χ***2 (3) = 28.26, P = 0000), demonstrating that the multivariate probit regression model produces more reliable results than the univariate Probit or logit model. The MVP results also suggest that the likelihood of adopting vegetable farming is 70.12%, followed by livestock rearing and crop-fruit production, which are 61.56% and 52.99%, respectively. In addition, the joint probability of success and failure to adopt all three UA practices was 40.52% and 17.54%, respectively (Table 4); indicating that more work has to be done to raise awareness, provide training, popularize, and create favorable conditions for urban farmers. Note that Stata software version 15 was used for the computation of all models.

**Table 3:** Correlation matrix of the urban agricultural practices from the MVP model.

Variables	Vegetable	Livestock	Crop-fruit
rho21	1.000		
rho31	0.0491***	1.000	
	(0.3364)		
rho32	0.0689**	−0.0207*	1.000
	(0.1770)	(0.6852)	

Likelihood ratio test of rho21 = rho31 = rho32 = 0: chi2(3) = 28.26.

Prob > chi2 = 0.0000.

**Table 4:** Multivariate probit estimations results of adoption urban agricultural practices.

	Vegetables		Livestock		Crop-fruit	
Variables	Coef. (SE)	ME	Coef. (SE)	ME	Coef. (SE)	ME
Sex	0.495***(0.169)	0.099	0.428*(0.249)	0.085	0.135 (0.153)	0.027
Age	−0.018**(0.008)	−0.004	−0.016 (0.012)	−0.003	−0.015**(0.007)	−0.003
Education	0.037*(0.019)	0.007	0.014 (0.027)	0.003	0.006 (0.017)	0.001
Occupation	−0.008 (0.190)	−0.002	0.185 (0.238)	0.037	−0.051 (0.161)	−0.010
Family size	−0.049 (0.043)	−0.010	−0.101 (0.078)	−0.020	0.056 (0.043)	0.011
Land size	1.350***(0.392)	0.269	0.798*(0.479)	0.159	0.279 (0.323)	0.056
Extension	0.144**(0.063)	0.029	0.066 (0.079)	0.013	0.062 (0.052)	0.012
Perception	−0.020 (0.251)	−0.004	8.326***(0.677)	0.662	0.645***(0.205)	0.129
Credit	0.965***(0.209)	0.192	0.761***(0.288)	0.152	−0.003 (0.171)	−0.001
Experience	0.014 (0.013)	0.003	0.045**(0.018)	0.009	0.019*(0.011)	0.004
PCMNTY	0.722***(0.209)	0.144	2.961***(0.341)	0.591	0.218 (0.189)	0.043
MDSTKM	0.069 (0.067)	0.014	0.209**(0.088)	0.042	0.127**(0.057)	0.025
Training	1.485***(0.209)	0.296	0.896***(0.278)	0.179	0.850***(0.192)	0.170
dNFINM	0.817***(0.300)	0.163	0.740*(0.420)	0.148	0.339 (0.278)	0.068
DRTO	0.224 (0.260)	0.045	0.082 (0.317)	0.016	−0.407*(0.223)	−0.081
Constant	−9.448***(3.372)		−11.118 (4.645)		−4.601 (3.067)	
Predicted probability		0.7012		0.6156		0.5299
Joint Probability (Success)				0.4052		
Joint Probability (Failure)				0.1754		
Draw Number (#)				100		
Observations				385		
Log Likelihood				−375.62		
Wald ***χ***2 (45)				711.07		
Prob > ***χ***2				0.000***		

According to the Simulated Maximum Likelihood estimation of the MVP results, household decisions to adopt livestock and vegetable practices, crop-fruit and vegetable practices, and crop-fruit and livestock practices are all positively and significantly interdependent. The correlation coefficients of the error terms show that diverse UA practices adopted by urban farmers are complimentary (positively correlated). This supports the assumption of interdependence between the different UA practices (Table 3). Since the parameters of the MVP model result cannot directly show the magnitude of the effect of an independent variable on the dependent variables, it is necessary to compute marginal effects (Rahman and Chima, 2016).

Results indicated that sex, age, education level, land size, extension contact, credit access, participation in community groups, participation in UA-related training, and log of non-farm income of the households had a significant influence on the adoption of vegetable production. On the other hand, adoption of livestock rearing was significantly affected by sex, land size, perception, credit access, farming experience, participation in community groups, market distance, participation in UA-related training, and log of non-farm income. Crop-fruit production practices are significantly influenced by age, perception, farming experience, market distance, training, and dependency ratio. Thus, the MVP analysis results conveyed that the decision to adopt each one of the UA practices is impacted by different factors and at various levels of significance by the same factor (Table 4).

The sex of the household head is positive and significantly influenced the adoption of vegetable and livestock production practices at 1% and 10% levels of significance, respectively. The positive and significant association implies that being female increases the probability of adopting vegetable and livestock farming practices by 9.9% and 8.5%, respectively. This means that female-headed households were more likely to adopt vegetable and livestock production practices compared to their counterpart, male-headed households. This result is in line with the findings of [19,39] who found that female-headed households are more likely to adopt UA practices than their male counterparts. This derives from their responsibility for household food provision and preparation. Nonetheless, it is clear that unpaid workers with a low opportunity cost, especially females, are relied upon to produce and market the vast majority of the sector’s output [42]. In the study areas, females were slightly more active in sowing, cultivation, harvesting, and livestock raring than males.

The age of the household head negatively and significantly affected both the vegetable and crop-fruit practices at a 5% significance level. The result unveils young household heads are more likely to adopt UA practices. This could be because young household heads better understand UA as the source of fresh and healthier food, value the economic returns from UA practices, and are associated with higher risk-taking behavior. The negative association implies that as the age of the household head increases by one unit, the probability of vegetable and crop-fruit practices adoption tended to decline by 0.4 and 0.3 percentage points, respectively. On the other hand, older people are associated with limited horizons in planning and are perceived as more risk-averse [43]. Moreover, older people have lower access to information, limit their movement from place to place, and become reluctant to adopt different UA practices. This may be because they are less receptive to new ideas and are less willing to take risks associated with innovations, as are the younger ones [44].

The household head's educational status is considered a crucial factor in enhancing the adoption of UA practices. It was assumed that formal or informal education would improve a household's awareness, knowledge, and competence about the importance of agricultural practices in urban areas [45]. Education helps to increase households' ability to access, perceive, and interpret innovative ideas. Results of the MVP model indicate that the education level (in terms of years of schooling) is positively and significantly related to the likelihood of vegetable practice adoption. The coefficient of education is positive, and this may be because better-educated farmers are more likely to participate in UA practices. A unit increase in the educational status of the household head increases the probability of vegetable practice adoption by 0.7%. This finding is in harmony with the findings of [45,46].

Land is the primary economic resource in urban areas, be it for industrial, residential, service, or agricultural activity, and hence, its availability may affect the adoption of UA practices. Accordingly, land size positively and significantly influenced vegetable and livestock practices. This showed households with relatively large land sizes are more likely to adopt vegetable and livestock practices. The model result indicates an increase in the land size by 1 ha would increase the likelihood of vegetable and livestock practices by about 26.9% and 15.9%, respectively. This result is consistent with the findings of [19,21], who underscore land as the main barrier to sustainable UA practice. Moreover, focused group discussants noted that urban land policy tends to favor individuals or groups who invest in small and medium-sized businesses other than UA, indicating that much work remains to be done to reduce UA's land constraint. Therefore, the government needs to ease its urban land use policy and encourage urban dwellers to engage in UA practices and maximize its benefits.

Access to extension services provides a platform for acquiring pertinent information, which encourages the adoption of agricultural practices. Access to information through extension services reduces disarray about the effectiveness of technology and, as a result, may shift an individual's opinion from entirely subjective to objective over time, encouraging adoption. According to the MVP model results, extension contact increased vegetable practice positively and significantly, suggesting that extension is crucial for UA practice. This study found that households with more extension contact are more likely to practice vegetable practice. The model also revealed that increasing the frequency of extension contact increased the likelihood of vegetable practice by 2.9%. The result is in line with the findings of [[47], [48], [49]]. This is because interacting with extension agents enhances the likelihood of obtaining up-to-date information on necessary agricultural technology. Urban agriculture is carried out under particular circumstances that need technology and organizational structures that differ from those used in rural agriculture, stressing the need to develop technical capacity and extension capabilities in most cities [50].

The comparative economic incentive essential to adopting UA practices has a significant and positive effect on the probability of adopting livestock and crop-fruit practices at a 1% significance level. Household heads who perceived the economic return from UA practices were more likely to adopt livestock and crop-fruit practices by 66.2% and 12.9% probability levels, respectively. Individuals, as rational decision-makers, seek to maximize return by allocating their resources, particularly land and labor, to higher economic return activities. Except for the initial cost, UA requires a small investment and monitoring costs in comparison to its economic return. Households may only accept agricultural technologies if they are aware of the economic benefits and inherent attributes of these technologies [51]. Other studies [52] reported similar findings.

When examining an individual's access to resources, one of the most important aspects is access to credit. It promotes technology adoption by reducing financial limitations and enhancing the risk-taking capability of households. Households with access to credit are less likely to sacrifice their agricultural needs for other expenditures [53]. The result of the MVP model revealed credit access has a positive and significant impact on vegetable and livestock practices at a 1% significance level. Households with access to credit are more likely to adopt vegetable and livestock practices than households without credit access at 15.2% and 12.2% probability levels, respectively. This is because credit access solves the cash constraints of poor urban farmers. The result is consistent with the findings of [54,55]. In addition, the findings from KIIs and FGD revealed that credit is limited to the UA sub-sector due to the associated risks, implying that local officials, financial institution decision-makers, and other relevant stakeholders should collaborate to alleviate the constraint and encourage urban dwellers to participate in UA practices widely.

Understanding the significance of people's long-term engagement in and experience with farming operations, interpersonal connections, and community services, and how they create the character of the community, is necessary for UA implementation. Mutual learning and experience sharing are essential components of UA practice if communities are to successfully implement bottom-up, community-led UA projects. Farming experience of the household head positively and significantly affected the livestock and crop-fruit practices adoption at a 5% and a 10% significance level, respectively. A one-year increase in farming experience reveals a 0.9% and 0.5% higher likelihood of adopting livestock and crop-fruit practices adoption, respectively. The assumption is that more experienced households can understand and identify changes related to farming practices easily. The results are consistent with the findings of [56,57].

For small-scale urban farmers, membership in a community group can be a channel for better access to extension services and the exchange of experiences and information with other households. The results of the MVP model revealed membership in a community group positively and significantly influenced both vegetable and livestock practices adoption at a 1% significance level. This shows members of a community group are 14.4% and 59.1% more likely to adopt vegetable and livestock practices than non-members. One plausible explanation for this could be that, in addition to the exchange of experiences and information, members have access to different seeds and medication at lower than market prices as subsided by the government through community groups. Numerous studies have also shown that membership increases access to information on new technologies, material inputs for technologies like fertilizers and pesticides, and credit for the purchase of inputs and the payment of hired labor [58]. The study findings of [55,59], and [60] are consistent with this result.

The MVP model result showed the distance from the market center of the household head positively and significantly affected the livestock and crop-fruit practices adoption at a 5% significance level. The positive sign of market distance from the market center indicates that households far away from the market center are more likely to adopt livestock and crop-fruit practices at a 4.2% and 2.5% probability levels, respectively than those who are located in the vicinity of the market centers. This could be because unlike in rural areas, households who reside in urban centers have better transportation facilities. Moreover, households who reside closer to the market center use the available land for other economic uses (such as cafes, restaurants, shops, etc.) and this situation changes as one goes far away from the market center. Hence, households who reside towards the periphery of the town/city allocated the available land better for UA practices. This finding is in harmony with the findings of [61].

One of the extension activities is training, through which households acquire useful skills and technical knowledge about improved technologies. It is an essential extension approach for encouraging household technology adoption. Participation in training has a positive and significant impact on the adoption of vegetable, livestock, and crop-fruit practices at a 1% significance level. This means participants of UA-related training are more likely to adopt vegetable, livestock, and crop-fruit practices at 29.6%, 17.9%, and 17% probability levels than non-participant households, respectively. This might be explained by the fact that families who have received training in any UA-related practice have a greater understanding of the advantages of the practices than non-trainers, which contributes to an increase in UA practice. The findings are consistent with previous research that indicated a link between training and the adoption of certain farming practices [62].

In many developing countries off-farm and non-farm income acts as an important strategy for overcoming credit constraints faced by farming households [63]. Income sources other than UA practices were expected to provide households with liquid capital for purchasing productivity-enhancing inputs such as improved seeds, fertilizers, and improved livestock breeds. Accordingly, income sources other than UA practices (log of non-farm income) of the respondents had a significant positive relationship at a 1% and a 10% level for vegetable and livestock practices adoption, respectively. This shows a 1% increase in the log of non-farm income of the household head increases the probability of vegetable and livestock practices adoption by 16.3% and 14.8%, respectively. The result is in line with the findings of [59,64].

The burden of social dependence on a household is measured using the dependency ratio, which mandates each individual of working age be able to support the existing dependents in the family. The dependency ratio of the household head negatively and significantly affected the crop-fruit practices adoption at a 10% significance level. This revealed that an increase in the number of dependents by one person decreases the likelihood of crop-fruit practice adoption by an 8.1% probability level. This conclusion might be connected to the notion that families with a higher dependence ratio place a greater responsibility on the actively working members of the urban farmers to supply food for the non-productive members. In such instances, it is critical to explore enhancing and restructuring current local labor-sharing mechanisms. On the other hand, the chance of adopting the crop-fruit practice decreased with the number of dependents living in the household, validating the intuitive notion that time spent caring for dependents diverts labor away from UA practices. The result is consistent with the findings of [39,65].

## 4 Conclusion and recommendations

To integrate UA practices as a livelihood strategy, various issues must be considered, including the associated institutional, political, technical, economic, social, and environmental motivations and limits. Data from 385 representative households were used to assess urban farmer’s decisions to adopt UA practices. Specifically, urban dwellers who practice vegetable, livestock, and crop-fruit farming in the Dire Dawa city administration and the east Haraghe zone of Oromia were studied using MVP modeling, which considers possible interdependence among their actions as individuals. The MVP model results show that factors such as sex, age, education, credit access, training, membership in community groups, participation in off-farm and non-farm activities, and land size have a statistically significant impact on vegetable practices adoption. Factors such as sex, perception, experience, credit access, training, membership in community groups, participation in off-farm and non-farm activities, and land size have a statistically significant impact on livestock practice adoption. While crop-fruit practice adoption is significantly influenced by factors such as age, dependency ratio, perception, and experience and variables that determine access to institutions, such as market distance and participation in UA-related training. In general, land shortage, poor access to information and agriculture extension services, and lack of credit availability are the major constraints influencing UA practices in the study areas.

To enhance UA practices policymakers and development practitioners can consider the following recommendations, which are based on the study's findings. The regional government, municipal agricultural development office, and donor agencies should work together with urban farmers to improve information access, accessibility and efficacy of extension contacts, and access to credit. They should also offer training and consulting services to encourage the adoption of UA practices. Moreover, the findings revealed that being female is more likely to adopt UA practices. Therefore, the town/city UA office needs to support women, particularly with the introduction of innovative agricultural technologies that require small land size and less input. Furthermore, it is also critical that local government offices, municipal administrations, and donor organizations collaborate to implement policies and initiatives that encourage UA practices. For example, to reduce land constraints, town and city municipals can create land inventories and distribution methods in their programs and offer formal assistance through collaborations.

## Funding

The authors declare that no funding was received for this manuscript.

## Data availability statement

Data will be made available on request.

## CRediT authorship contribution statement

**Gebregziabher Nigus:** Writing – original draft, Methodology, Investigation, Formal analysis, Data curation, Conceptualization. **Mengistu Ketema:** Methodology. **Jema Haji:** Methodology. **Million Sileshi:** Methodology.

## Declaration of competing interest

The authors declare that they have no known competing financial interests or personal relationships that could have appeared to influence the work reported in this paper.
