# Supplementary material for: Determinants of adoption of urban agricultural practices in eastern Haraghe zone of Oromia region and Dire Dawa City administration, eastern Ethiopia
Source: Heliyon. 2024 Feb 20;10(4):e26758. doi: 10.1016/j.heliyon.2024.e26758 (PMC10906398; doi:10.1016/j.heliyon.2024.e26758)
Supplement: Multimedia component 1 [file mmc1.docx]

**Urban Agricultural Practices, Market Participation Decisions, and its Impact on Households’ Welfare in Eastern Ethiopia**

Ph.D. Dissertation Research Survey Questionnaire

Postgraduate Program Directorate, Haramaya University

**General Direction to Enumerators**

1) Please introduce yourself before starting the interview.

2) Inform that the purpose is to acquire first-hand information that will only be utilized for scientific writing on the aforementioned topic.

3) In cases where precise measurements are not available, kindly exercise caution in estimating.

4) When your interview is over, don't forget to say "thank you."

Thank you for your kind co-operation.

**Thesis the objectives**

The general objective of the study focuses on urban agricultural practice and the role it plays in influencing urban farmers’ decision-making process and their welfare in some selected towns and (or) cities of the Eastern Hararghe zone of Oromia and Dire Dawa city administration, Ethiopia. The specific objectives are:

1) Determine factors affecting the adoption of urban agricultural practices;

2) Investigate the intensity of urban agriculture market participation and its determinants; and

3) Evaluate the welfare impact of urban agricultural practices adoption.

**Household ID**

**Date: ____________________**

Questionnaire ID (001-385): ________________________

District: ___________________ Town/City: ________________ Kebele: ________________

Enumerator’s name (code): ________________________ Signature: _____________

**Section One: Characteristics of the Household**

1. Sex of the household head? 1) Male 2) Female

2. Age of the household head (in years)? _________________

3. Marital status of the household head?

1) Single 2) Married 3) Divorced 4) Widowed

4. Household head’s years of schooling in number? __________________________

5. Household size in number? ­­­­­­­­­­­­­____________________________

6. Dependency ratio?

| No | Household age category | Dependency ratio |
| --- | --- | --- |
| 1 | Less than 10 years olds |  |
| 2 | A male children between 10-13 years olds |  |
| 3 | A female children between 10-13 years olds |  |
| 4 | A male member between 14 and 65 years olds |  |
| 5 | A female member between 14 and 65 years olds |  |
| 6 | Family member above 65 years olds |  |

6. Occupation of the household head?

1) Civil servant 2) Daily worker 3) Merchant 4) Urban farmer

5) Other, specify _____________________

**Section Two: Information on Urban Agriculture**

1. Do you practice urban agriculture (UA)? 1) Yes 2) No

2. If the answer to (Q.1) is yes, for how long have you been practicing UA (in Years)? ______________________________

3. What type of urban agriculture do you practice?

1) Vegetables 2) Livestock 3) Crops-fruits 4) Other, specify _____________

4. Number of family members participating in urban agricultural activities?

1) Male: _________ 2) Female: ___________

5. What is the tenure system of your farm?

1) Own Land 2) Family Land 3) Rented Land 4) other, specify _________

6. Size of land in hectares? ___________________________

7. What is the reason for you to start urban agricultural practices?

1) Household food shortage 2) Income generation 3) Medicine

4) Unemployment 5) other, specify **________________________________**

8. Do you think UA is beneficial to the livelihood of your family?

1) Yes 2) No

9. What are the sources of labor for UA production?

1) Family labor 2) Relatives 3) Hired labor

4) Other, specify _________________________________________________________

10. What are the associated problems?

| **No** | **Problems** | **Tick mark** |
| --- | --- | --- |
| 1 | Lack of land for production |  |
| 2 | Lack of inputs (seeds, breeds, fertilizer, credit facilities, etc.) |  |
| 3 | Low market access (low price, low demand of products, etc.) |  |
| 4 | Threat of pollution |  |
| 5 | Water shortage |  |
| 6 | Pest and disease stress |  |
| 7 | Poor access to information and agriculture extension services |  |
| 8 | Low value chain |  |

**Section Three: Vegetables and Crop-fruits Production**

1. Do you grow vegetables and/or crop-fruits? 1) Yes 2) No

2. Where do you grow the vegetables and/or crop-fruits?

1) In backyards 2) In urban fringe areas 3) Balkonies/walls

4) In open space 5) Roadsides 6) other, specify ______________

3. Are seeds and seedlings easily accessible? 1) Yes 2) No

4. Where do you get seeds and seedlings for your UA practice?

1) Cooperatives 2) Private suppliers 3) Local town/city nursery

4) Other, specify___________________________________________________________

5. Quantity of vegetables produced in a year (in Kg)? ___________________________

6. Approximately how many quantities do you sale annually (in Kg)? ___________________

7. What are the major crop and fruit types you have been using to your land?

| **No** | **Crop grown** | **Unit** | **Average yield/year** | **Price per unit** | **Average value** |
| --- | --- | --- | --- | --- | --- |
| 1 | Maize | Kg |  |  |  |
| 2 | Sorghum | Kg |  |  |  |
| 3 | Teff | Kg |  |  |  |
| 4 | Wheat | Kg |  |  |  |
| 5 | Haricot bean | Kg |  |  |  |
| 6 | Pea | Kg |  |  |  |
| 7 | Barley | Kg |  |  |  |
| 8 | Chickpea | Kg |  |  |  |
| 9 | Papaya | Kg |  |  |  |
| 10 | Mango | Kg |  |  |  |
| 11 | Avocado | Kg |  |  |  |
| 12 | Banana | Kg |  |  |  |
| 13 | Orange | Kg |  |  |  |
| 14 | Chat | Kg |  |  |  |
| 14 | Lemon | Kg |  |  |  |
| 16 | Seedlings (Trees) | No |  |  |  |
|  |  |  |  |  |  |
|  |  |  |  |  |  |

8. What are the major vegetable types you have been using to your land?

| **No** | **Vegetables and fruits grown** | **Unit** | **Frequency of harvest/year** | **Average yield** | **Price per unit** | **Average value** |
| --- | --- | --- | --- | --- | --- | --- |
| 1 | Pepper | Kg |  |  |  |  |
| 2 | Carrot | Kg |  |  |  |  |
| 3 | Potato | Kg |  |  |  |  |
| 4 | Tomato | Kg |  |  |  |  |
| 5 | Cabbage | Kg |  |  |  |  |
| 6 | Beet root | Kg |  |  |  |  |
| 7 | Onions | Kg |  |  |  |  |
| 8 | Lettuce | Kg |  |  |  |  |
| 9 |  |  |  |  |  |  |
| 10 |  |  |  |  |  |  |

**Section Four: Livestock production**

1. Do you raise livestock? 1) Yes 2) No

2. For what purpose do you raise livestock?

1) Own-consumption 2) Sale 3) Manure

4) Transport 5) other, specify ___________________

3. Are livestock breeds easily accessible? 1) Yes 2) No

4. Is there shortage of livestock feed in your town/city? 1) Yes 2) No

5. From where do you get feed for your livestock?

1) Purchase 2) Own production 3) other, specify____________________

6. For what purpose do you use livestock dung?

1) As manure 2) Fuel wood 3) No use 4) Other, specify _________________

7. Indicate the type of livestock you rare in the household?

| No | Category | Total | TLU |
| --- | --- | --- | --- |
| 1 | Ox |  |  |
| 2 | Cow |  |  |
| 3 | Calf |  |  |
| 4 | Goat young |  |  |
| 5 | Goat adult |  |  |
| 6 | Sheep young |  |  |
| 7 | Sheep adult |  |  |
| 8 | Hen (Broilers) |  |  |
| 9 | Hen (Laying hens) |  |  |
| 10 | Donkey (Young/Adult) |  |  |
| 11 | Camel |  |  |
| 12 | Mule |  |  |

**Section Five: Household Income**

1. Do you think UA has improved the household's income? 1) Yes 2) No

2. If the answer to (Q.1) is yes, would you tell me the monthly income obtained from UA? ____________________________________________

3. Does the income you generated improved your living condition? 1) Yes 2) No

4. Cash income from crops, vegetables, and/or fruits sale in 2021/22 (2014 E.C)

| **No** | | **List of crops/Vegetables/ fruits grown** | **Unit** | **Amount produced** | **Amount Consumed** | **Quantity Sold** | **Price per unit** | **Annual income** |
| --- | --- | --- | --- | --- | --- | --- | --- | --- |
| 1 | Maize | | Kg |  |  |  |  |  |
| 2 | Haricot beans | | Kg |  |  |  |  |  |
| 3 | Tomatoes | | Kg |  |  |  |  |  |
| 4 | Onions | | Kg |  |  |  |  |  |
| 5 | Cabbage | | Kg |  |  |  |  |  |
| 6 | Carrot | | Kg |  |  |  |  |  |
| 7 | Oranges | | Kg |  |  |  |  |  |
| 8 | Mangoes | | Kg |  |  |  |  |  |
| 9 | Bananas | | Kg |  |  |  |  |  |
| 10 |  | |  |  |  |  |  |  |
| 11 |  | |  |  |  |  |  |  |
| 12 |  | |  |  |  |  |  |  |
|  | Total | |  |  |  |  |  |  |

5. Livestock ownership and cash income from livestock sales in 2021/22 (2014 E.C)?

| **No** | **Type of Livestock** | **Total number Owned** | **Total number Sold** | **Price (Birr)** | **Total Value** |
| --- | --- | --- | --- | --- | --- |
| 1 | Cows |  |  |  |  |
| 2 | Oxen |  |  |  |  |
| 3 | Heifers |  |  |  |  |
| 4 | Calves |  |  |  |  |
| 5 | Bulls |  |  |  |  |
| 6 | Sheep |  |  |  |  |
| 7 | Chicken |  |  |  |  |
| 8 | Donkey |  |  |  |  |
| 9 | Horse |  |  |  |  |
| 10 | Goats |  |  |  |  |
|  | Others |  |  |  |  |
|  | Grand total |  |  |  |  |

6. Cash income from livestock products sale in 2021/22 (2014 E.C)?

| **No** | **Type of product** | **Unit** | **Quantity produced/Month** | **Quantity Sold /Month** | **Unit Price** | **Total Value** |
| --- | --- | --- | --- | --- | --- | --- |
| 1 | Milk | Liter |  |  |  |  |
| 2 | Eggs | No |  |  |  |  |
| 3 | Butter | kg |  |  |  |  |
| 4 | Cheese | kg |  |  |  |  |
| 5 | Honey | kg |  |  |  |  |
| 6 |  |  |  |  |  |  |
| 7 |  |  |  |  |  |  |
|  | Total |  |  |  |  |  |

7. Do you or your family members participate in some additional income generating activities (off-Farming and non-farm activities) in the year 2021/22 (2014 E.C)?

1) Yes 2) No

8. Income earned from the following activities

| **No** | **Non-farm income**  **Sources** | **Number of participants** | **Income per month** | **Total annual income** |
| --- | --- | --- | --- | --- |
| 1 | Wage (Salary) |  |  |  |
| 2 | Selling local drink |  |  |  |
| 3 | Pottery |  |  |  |
| 4 | Handicraft |  |  |  |
| 5 | Weaving |  |  |  |
| 6 | Pension payments |  |  |  |
| 7 | Ceramic |  |  |  |
| 8 | Carpenter |  |  |  |
| 9 | Rent from assets |  |  |  |
| 10 | Trading |  |  |  |
| 11 | Clothes making |  |  |  |
| 12 | Driver |  |  |  |
| 13 | Remittance |  |  |  |
|  | Other |  |  |  |

9. For what purposes do you spend the income you generate from the UA?

1) For different family expenses 2) For students school expenses

3) For savings 4) For farm expansion

5) For other, specify ______________________________________________________

**Section Six: Institutional Support**

1. Do you have extension contact in relation to UA practices/marketing in 2021/22 (2014 E.C)?

1) Yes 2) No

2. How many times do you meet the extension workers? ____________

3. Do you have access to credit for your farming operation? 1) Yes 2) No

4. If the answer to (Q.3) is yes, from where and how much do you obtain?

| **No** | **Source of credit** | **Amount (Birr)** | **Interest rate** | **Remarks** |
| --- | --- | --- | --- | --- |
| 1 | Friends/relatives |  |  |  |
| 2 | Microfinance |  |  |  |
| 3 | Cooperative/union |  |  |  |
| 4 | Bank |  |  |  |
| 5 | Iqub |  |  |  |
| 6 | Iddir |  |  |  |
| 7 | NGOs |  |  |  |
| 8 |  |  |  |  |
| 9 |  |  |  |  |
|  | **Total** |  |  |  |

5. If the answer to (Q.3) is yes, for what purpose do you use it? (Multiple answer possible)

1) To buy agricultural inputs 2) To buy livestock 3) To pay school fee

4) To cover house hold expenditure 5) other, specify __________________________

6. If the answer to (Q.3) is No, what are your sources of finance for farming operation?

1) Crop sales 2) Livestock sales 3) Non-farm activities

4) Other, specify __________________________________________________________

7. Have you ever received any UA related training? 1) Yes 2) No

8. If the answer to (Q.7) is yes, who are the organizers of the training?

1) GO 2) NGO 3) other, specify____________________

9. Do you think that the training was helpful and solved your practical problems?

1) Yes 2) No

10. If the answer to (Q.9) is no, why? __________________________________________

11. Are you a member of any community group (local organization, Idir, Iqub, etc.)?

1) Yes 2) No

12. What is/are the benefit/s you get from the group?

1) Market together 2) Information access 3) Credit access

4) Acquire inputs 5) other, specify ________________________________

13. Type of information or services you need in UA practice and marketing?

| No | Extension service required | Rank |
| --- | --- | --- |
| 1 | Cultivation |  |
| 2 | Market information |  |
| 3 | Harvesting |  |
| 4 | Processing |  |
| 5 | Other specify |  |

**Section Seven: Marketing service and Price**

1. Do you sale any of your products in a market? 1) Yes 2) No

2. If the answer to (Q.1) is yes, where do you sale most of your UA produces? _________

1) Farm gate 2) Urban/Local market 3) Super market 4) Export

5) Other, specify _______________

3. What is the reason you selected to sale to the one indicated in Q.2?

1) Pays high price than others 2) Frequent purchase

3) Low transportation cost 4) other, specify __________________________

4. Major buyers of UA products: (Multiple answers is possible)

| **No** | **Place** | **Tick where appropriate** |
| --- | --- | --- |
| 1 | Processors |  |
| 2 | Wholesalers |  |
| 3 | Retailers |  |
| 4 | Consumers |  |
| 5 | Cooperatives |  |
| 6 | Other |  |

5. How far is the market center from your house (in Kms)? _____________________________

6. Do you have access to market information? 1) Yes 2) No

7. What are your sources of information?

| **No** | **Sources** | **Tick where appropriate** | **Reason** |
| --- | --- | --- | --- |
| 1 | Public administration |  |  |
| 2 | Extension agents |  |  |
| 3 | Farmer Cooperatives or groups |  |  |
| 4 | Co-farmers |  |  |
| 5 | NGOs |  |  |
| 6 | Media: Radio/TV/ Mobile phone |  |  |
| 7 | Market |  |  |
| 8 | DA |  |  |

8. What are the costs involved when selling UA products to the market?

1) Transportation 2) Dealers/brokers 3) Tax

4) Other, specify _____________________________________

9. How do you make decisions about when to sale your UA output?

1) Maturity 2) Fear of drought 3) Market price

4) Demand for consumption 5) other, specify _________________________

10. Do you face any difficulty in finding buyers when you want to sale?

1) Yes 2) No

11. If the answer to (Q.10) is yes, is this due to:

1) Inaccessibility of market 2) Lack of information 3) Low price offered

4) Other, specify _________________________________________________________

12. What do you do if you do not get the expected price for your UA product?

1) Take it back home 2) Sale at a lower price

3) Take it to another market on the same day 4) Sale it on another market day

13. Are you beneficiary of this market linkage program? 1) Yes 2) No

14 How do you evaluate the UA product market access in your town/city?

1) Poor 2) Medium 3) Good

15. Do you think poor market access affected UA production? 1) Yes 2) No

16. Do you think that the market for UA output is right enough? 1) Yes 2) No

17. List what you consider to be the major problems you face in marketing your products?

| **No** | **Problems** | **Tick mark** |
| --- | --- | --- |
| 1 | Lack of saleable surplus |  |
| 2 | Low price of agricultural produce |  |
| 3 | Low bargaining power |  |
| 4 | Inadequate access to market for their products |  |
| 5 | Low marketing awareness |  |
| 6 | Poor access to information and agriculture extension services |  |
| 7 | Low value chain |  |
| 9 | Others |  |

**Section Eight: Household Expenditure**

1. From where do you get food for your family? (Multiple answer possible)

1) Own produce 2) Food aid 3) Purchase 4) Gift

5) Borrow from relatives 6) Share from relatives 7) Borrow from neighbors

8) Other, specify ______________________

2. How much money do you spend per month for your livelihood? ______________________

3. Household food consumption expenditure in 2021/22 (2014 E.C)

| Food type | Unit | Own production | | | Purchased | | | Receivable in kind, gift, aid, etc. | |
| --- | --- | --- | --- | --- | --- | --- | --- | --- | --- |
|  |  | Quan- tity | Unit price (Birr) | Total expenditure | Quan- tity | Unit price (Birr) | Total expenditure | Quan- tity | Source |
| **Cereals** | | | | | | | | | |
| Maize | Kg |  |  |  |  |  |  |  |  |
| Sorghum | Kg |  |  |  |  |  |  |  |  |
| Wheat | Kg |  |  |  |  |  |  |  |  |
| Teff | Kg |  |  |  |  |  |  |  |  |
|  |  |  |  |  |  |  |  |  |  |
| **Pulses** | | | | | | | | | |
| Faba bean | Kg |  |  |  |  |  |  |  |  |
| Lentils | Kg |  |  |  |  |  |  |  |  |
| Haricot beans | Kg |  |  |  |  |  |  |  |  |
|  |  |  |  |  |  |  |  |  |  |
|  |  |  |  |  |  |  |  |  |  |
| Vegetables | | | | | | | | | |
| Tomatoes | Kg |  |  |  |  |  |  |  |  |
| Onions | Kg |  |  |  |  |  |  |  |  |
| Cabbage | Kg |  |  |  |  |  |  |  |  |
| Spinach | Kg |  |  |  |  |  |  |  |  |
| Carrot | Kg |  |  |  |  |  |  |  |  |
| Pumpkin | Kg |  |  |  |  |  |  |  |  |
|  |  |  |  |  |  |  |  |  |  |
| Food type | Unit | Own production | | | Purchased | | | Receivable in kind, gift, aid, etc. | |
|  |  | Quan- tity | Unit price (Birr) | Total expenditure | Quan- tity | Unit price (Birr) | Total expenditure | Quan- tity | Source |
| Cucumber | Kg |  |  |  |  |  |  |  |  |
| Pepper | Kg |  |  |  |  |  |  |  |  |
| Potatoes | Kg |  |  |  |  |  |  |  |  |
| Garlic | Kg |  |  |  |  |  |  |  |  |
|  |  |  |  |  |  |  |  |  |  |
|  |  |  |  |  |  |  |  |  |  |
| **Fruits** | | | | | | | | | |
| Oranges | Kg |  |  |  |  |  |  |  |  |
| Mangoes | Kg |  |  |  |  |  |  |  |  |
| Pineapple | Kg |  |  |  |  |  |  |  |  |
| Bananas | Kg |  |  |  |  |  |  |  |  |
| Apple | Kg |  |  |  |  |  |  |  |  |
| Guava | Kg |  |  |  |  |  |  |  |  |
| Papaya | Kg |  |  |  |  |  |  |  |  |
| Lemon | Kg |  |  |  |  |  |  |  |  |
| **Animal Products** | | | | | | | | | |
| Cattle meat | Kg |  |  |  |  |  |  |  |  |
| Camel meat | Kg |  |  |  |  |  |  |  |  |
| Goat meat | Kg |  |  |  |  |  |  |  |  |
| Sheep meat | Kg |  |  |  |  |  |  |  |  |
| Chicken | Kg |  |  |  |  |  |  |  |  |
| Fish | Kg |  |  |  |  |  |  |  |  |
| Egg | No |  |  |  |  |  |  |  |  |
| Cow Milk | Liter |  |  |  |  |  |  |  |  |
| Cheese | Kg |  |  |  |  |  |  |  |  |
| Butter | Kg |  |  |  |  |  |  |  |  |
| Honey | Kg |  |  |  |  |  |  |  |  |
| Camel milk | Liter |  |  |  |  |  |  |  |  |
|  |  |  |  |  |  |  |  |  |  |
|  |  |  |  |  |  |  |  |  |  |
| **Beverages and Energy** | | | | | | | | | |
| Tea | Kg |  |  |  |  |  |  |  |  |
| Coffee | Kg |  |  |  |  |  |  |  |  |
| Soft drinks | Liter |  |  |  |  |  |  |  |  |
| Local beer | No |  |  |  |  |  |  |  |  |
| Bottled beer | No |  |  |  |  |  |  |  |  |
| Wine | Liter |  |  |  |  |  |  |  |  |
| Water | Liter |  |  |  |  |  |  |  |  |
| Chat | Kg |  |  |  |  |  |  |  |  |
| Cigarettes | Pack |  |  |  |  |  |  |  |  |
|  |  |  |  |  |  |  |  |  |  |
|  |  |  |  |  |  |  |  |  |  |
| **Fats, oils, and others** | | | | | | | | | |
| Edible oil | Liter |  |  |  |  |  |  |  |  |
| Bread | No |  |  |  |  |  |  |  |  |
| Rice | Kg |  |  |  |  |  |  |  |  |
| Macaroni | Kg |  |  |  |  |  |  |  |  |
| Pasta | Kg |  |  |  |  |  |  |  |  |
| Sugar | Kg |  |  |  |  |  |  |  |  |
| Salt | Kg |  |  |  |  |  |  |  |  |
| Ginger | Kg |  |  |  |  |  |  |  |  |
|  |  |  |  |  |  |  |  |  |  |
|  |  |  |  |  |  |  |  |  |  |

4. Household non-food consumption expenditure in 2021/22 (2014 E.C)

| No | Type of non-food items | Total Expenditures (Birr) |
| --- | --- | --- |
| 1 | Kerosene |  |
| 2 | Soap |  |
| 3 | Clothes and Shoes |  |
| 4 | Education fee |  |
| 5 | Transportation and communication |  |
| 6 | Medication cost (human and livestock) |  |
| 7 | House rent |  |
| 8 | Housing (e.g. for iron-sheet cover) |  |
| 9 | Farm implements (e.g. spade, sickle, axe, hoes, pump, etc.) |  |
| 10 | Durables (radio, bed, mattress, mobile, etc.) |  |
| 11 | Government tax |  |
| 12 | Religious and cultural expense |  |
|  | Other |  |
|  |  |  |
|  |  |  |

5. Mention types of food items consumed in the household by recalling seven days back

| Food type | Unit | Consumed food items | | | | | | | |
| --- | --- | --- | --- | --- | --- | --- | --- | --- | --- |
|  |  | Enjera | Bread /Kitta | Lafiso | Porri-dge | Cheche-bsa | Roasted (Nifro) | Other | Total |
| **Cereals** | | | | | | | |  |  |
| Maize | Kg |  |  |  |  |  |  |  |  |
| Sorghum | Kg |  |  |  |  |  |  |  |  |
| Wheat | Kg |  |  |  |  |  |  |  |  |
| Teff | Kg |  |  |  |  |  |  |  |  |
| **Vegetables** | | **Amount** |  |  |  |  |  |  |  |
| Tomatoes | Kg |  |  |  |  |  |  |  |  |
| Onions | Kg |  |  |  |  |  |  |  |  |
| Cabbage | Kg |  |  |  |  |  |  |  |  |
| Spinach | Kg |  |  |  |  |  |  |  |  |
| Carrot | Kg |  |  |  |  |  |  |  |  |
| Pumpkin | Kg |  |  |  |  |  |  |  |  |
| Cucumber | Kg |  |  |  |  |  |  |  |  |
| Pepper | Kg |  |  |  |  |  |  |  |  |
| Potatoes | Kg |  |  |  |  |  |  |  |  |
| Garlic | Kg |  |  |  |  |  |  |  |  |
|  |  |  |  |  |  |  |  |  |  |
| **Fruits** | | |  |  |  |  |  |  |  |
| Oranges | Kg |  |  |  |  |  |  |  |  |
| Mangoes | Kg |  |  |  |  |  |  |  |  |
| Pineapple | Kg |  |  |  |  |  |  |  |  |
| Bananas | Kg |  |  |  |  |  |  |  |  |
| Apple | Kg |  |  |  |  |  |  |  |  |
| Guava | Kg |  |  |  |  |  |  |  |  |
|  |  |  |  |  |  |  |  |  |  |
| **Animal Products** | | |  |  |  |  |  |  |  |
| Cattle meat | Kg |  |  |  |  |  |  |  |  |
| Camel meat | Kg |  |  |  |  |  |  |  |  |
| Goat meat | Kg |  |  |  |  |  |  |  |  |
| Sheep meat | Kg |  |  |  |  |  |  |  |  |
| Chicken | Kg |  |  |  |  |  |  |  |  |
| Fish | Kg |  |  |  |  |  |  |  |  |
| Egg | No |  |  |  |  |  |  |  |  |
| Cow Milk | Liter |  |  |  |  |  |  |  |  |
| Cheese | Kg |  |  |  |  |  |  |  |  |
| Butter | Kg |  |  |  |  |  |  |  |  |
| Honey | Kg |  |  |  |  |  |  |  |  |
| Camel milk | Liter |  |  |  |  |  |  |  |  |
|  |  |  |  |  |  |  |  |  |  |
| **Pulses and Oil crops** | | |  |  |  |  |  |  |  |
| Faba bean | Kg |  |  |  |  |  |  |  |  |
| Lentils | Kg |  |  |  |  |  |  |  |  |
| Haricot beans | Kg |  |  |  |  |  |  |  |  |
|  |  |  |  |  |  |  |  |  |  |
| **Beverages and Energy** | | |  |  |  |  |  |  |  |
| Tea | Kg |  |  |  |  |  |  |  |  |
| Coffee | Kg |  |  |  |  |  |  |  |  |
| Soft drinks | Liter |  |  |  |  |  |  |  |  |
| Local beer | No |  |  |  |  |  |  |  |  |
| Bottled beer | No |  |  |  |  |  |  |  |  |
| Wine | Liter |  |  |  |  |  |  |  |  |
| Water | Liter |  |  |  |  |  |  |  |  |
| Chat | Kg |  |  |  |  |  |  |  |  |
| Cigarettes | Pack |  |  |  |  |  |  |  |  |
|  |  |  |  |  |  |  |  |  |  |
| **Fats, oils, and others** | | |  |  |  |  |  |  |  |
| Edible oil | Liter |  |  |  |  |  |  |  |  |
| Bread | No |  |  |  |  |  |  |  |  |
| Rice | Kg |  |  |  |  |  |  |  |  |
| Macaroni | Kg |  |  |  |  |  |  |  |  |
| Pasta | Kg |  |  |  |  |  |  |  |  |
| Sugar | Kg |  |  |  |  |  |  |  |  |
| Salt | Kg |  |  |  |  |  |  |  |  |
| Ginger | Kg |  |  |  |  |  |  |  |  |

**Thank you for your cooperation.**
